# Proof of concept: a spatial modular small-world self-organises by adaptive rewiring

**DOI:** 10.1186/1471-2202-16-S1-P272

**Published:** 2015-12-18

**Authors:** Nick Jarman, Chris Trengove, Erik Steur, Ivan Tyukin, Cees van Leeuwen

**Affiliations:** 1 Perceptual Dynamics Laboratory, University of Leuven, Leuven, Flemish Brabant, B3000, Belgium; 2 Department of Mathematics, University of Leicester, Leicester LE1 7RH, UK; 3 Saint-Petersburg State Electrotechnical University, Saint-Petersburg, Saint Petersburg 197376, Russia

## 

A small-world network is a network that reconciles two opposing properties, segregation and integration. It is this reconciliation that gives rise to the impressive information processing capacity of the human brain; segregation provides a platform for information processing, whilst integration provides for the fast transmission of information. However, the connectivity structure of the brain is not static [1]; it changes on multiple time-scales; on a relatively fast time-scale, synaptic plasticity takes place, whilst on a slower time-scale there is rewiring of brain connectivity through growth of axons and dendrites. This structural plasticity depends on the even faster time-scale of neural activity. But the relationship is symbiotic: patterns of synchronous activity are, of necessity, mediated by the brain connectivity structure. Gong & van Leeuwen [2] showed that rewiring of an initially random network - adaptive rewiring - in a model of spontaneous cortical activity gives rise to a particular type of network connectivity structure: a modular small-world. In order to improve the applicability of such a model to the cortex, spatial characteristics of cortical connectivity need to be respected. For this purpose we consider networks endowed with a metric by embedding them into a physical space. Such spatial constraints may represent wiring and metabolic costs in the brain. We provide an adaptive rewiring model with a spatial distance function and a corresponding spatially local rewiring bias [3].

**Figure 1:**
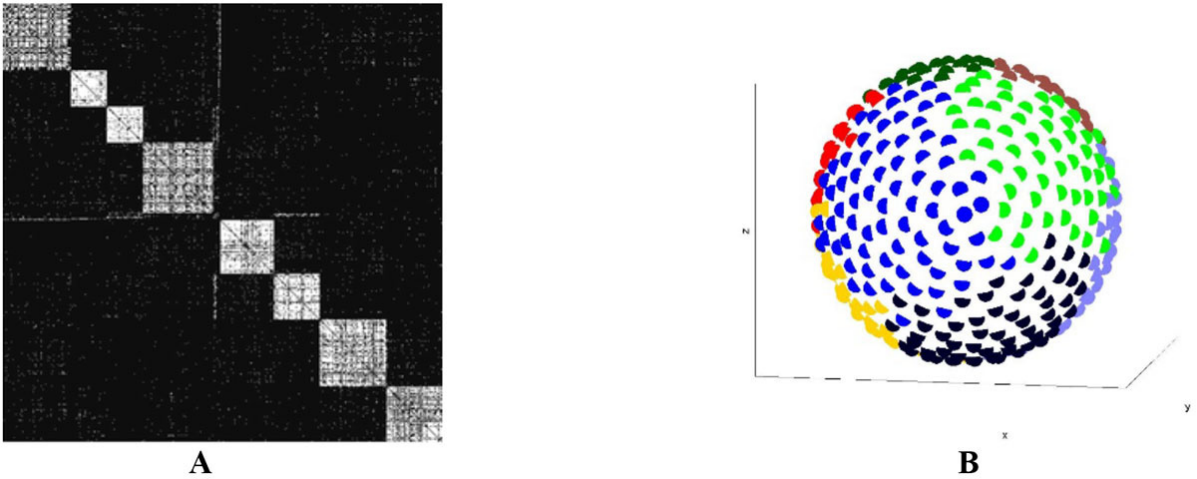
**A, Network adjacency matrix organised to optimise visual presentation of modular structure**. **B**, Units on the sphere colour-coded to identify distinct modules.

## Conclusion

The resulting rewiring scenarios showed a spatial layout of the connectivity structure, in which topologically segregated modules correspond to spatially segregated regions, and these regions are linked by long-range connections (see Figure 1, A and B). Greater realism and increased efficiency and robustness of the symbiosis of activity and structure is achieved compared to non-spatial adaptive rewiring. Thus, the principle of locally biased adaptive rewiring may explain both the topological connectivity structure and spatial distribution of connections between neuronal units in a large-scale cortical architecture.
